# Structure-Property Relationships Governing Encapsulation and Release of Antibiotics from Calcium–Alginate Hydrogels

**DOI:** 10.3390/gels12070636

**Published:** 2026-07-16

**Authors:** İbrahim Hebip, İrem Toprakçı, Rabia Nur Bozkurt, Ebru Kurtulbaş, Selin Şahin

**Affiliations:** 1 Department of Chemical Engineering, Faculty of Engineering, Istanbul University-Cerrahpaşa, Avcılar, 34320 Istanbul, Türkiyeirem.toprakciyuksel@iuc.edu.tr (İ.T.); rabia.bozkurt@istun.edu.tr (R.N.B.); ebru.kurtulbas@iuc.edu.tr (E.K.); 2 Department of Chemical Engineering, Faculty of Engineering and Natural Sciences, Istanbul Health and Technology University, Beyoğlu, 34440 Istanbul, Türkiye

**Keywords:** calcium-alginate hydrogels, Ionic crosslinking, drug-polymer interactions, diffusion-controlled transport, pH-responsive release

## Abstract

Understanding mass transport of structurally different drugs within ionically crosslinked hydrogel networks remains an important challenge in polymer-based delivery systems. In this study, hydrophilic amoxicillin (AMOX) and amphiphilic doxycycline (DOX) were encapsulated into calcium–alginate beads, respectively. A three-factor and three-level Box–Behnken design was utilized to examine the influences of alginate concentration (2–5%, *w*/*v*), CaCl_2_ concentration (1–3%, *w*/*v*), and gelation time (15–45 min) on encapsulation efficiency (EE). EE exhibited considerable variability for both AMOX (10–86%) and DOX (10–63%). Optimal EE values were achieved at almost 3.5% alginate and 3% CaCl_2_. The optimized gelation times differed between AMOX (45 min) and DOX (15 min), which is likely associated with differences in their physicochemical properties, although additional intermediate gelation times could further refine the optimal conditions. ANOVA identified CaCl_2_ concentration and the quadratic effect of alginate as the most influential parameters. Furthermore, both models demonstrated robust predictive capability (R^2^ > 0.98). In vitro release experiments demonstrated minimal drug diffusion in simulated gastric fluid (SGF) and significantly accelerated release in simulated intestinal fluid (SIF). These findings indicate a pH-responsive release behavior under simulated gastrointestinal conditions. The release profile was best represented by Higuchi and Korsmeyer–Peppas kinetic models. SEM and optical microscopy revealed uniform spherical beads with drug-dependent microstructural differences: hydrophilic AMOX produced smoother, wrinkled surfaces, whereas amphiphilic DOX induced localized cracking and heterogeneous microdomains. Furthermore, DLS and zeta potential measurements of the released fractions indicated nanoscale particle populations (≈190–225 nm) with moderate negative surface charge (≈−21 mV), suggesting stable colloidal dispersion during intestinal-phase release.

## 1. Introduction

Alginate is a linear anionic polysaccharide made up of β-D-mannuronic (M) and α-L-guluronic (G) acid residues organized in different block sequences [1]. Its capacity to build three-dimensional networks via ionic crosslinking with divalent or trivalent cations (Ca^2+^, Cu^2+^, Sr^2+^, and Al^3+^) makes it one of the most versatile biopolymers employed in colloid and interface science [2]. Calcium ions interact with G-blocks to form egg-box structures, causing hydrogel matrices characterized by adjustable network density, mechanical strength, and diffusional paths [3]. The physicochemical properties allow alginate hydrogels to behave as colloidal soft materials, characterized by distinct interfacial microdomains that regulate transport at the polymer-solution interface [4]. Calcium–alginate hydrogels’ ionically crosslinked structure also provided environmental adaptability in terms of pH [5]. Alginate hydrogels are valuable as oral matrices because of their pH-dependent interfacial transitions, which protect encapsulated molecules in harsh gastric conditions while facilitating regulated release in intestinal environments [6]. One of the goals in colloid and hydrogel science is to comprehend the effects of the formulation factors on the interfacial transitions and transport channels.

Earlier studies generally reported that the encapsulation and release behavior of alginate hydrogels depends strongly on factors such as alginate concentration, CaCl_2_ concentration, and gelation time [7,8,9,10]. Statistical experimental design approaches, such as response surface methodology (RSM), are powerful tools for quantifying the mechanistic influence of these variables on colloidal network formation and transport behavior [7].

Amoxicillin (AMOX) and doxycycline (DOX) were chosen as model compounds due to their representation of two structurally and physicochemically diverse families of commonly utilized oral antibiotics. AMOX is a hydrophilic β-lactam with relatively low molecular weight, whereas DOX is a more lipophilic tetracycline derivative with larger molecular size and stronger hydrophobic domains [11,12]. Therefore, evaluating their encapsulation and release independently within the same colloidal platform provides a meaningful framework to examine how molecular features govern interfacial interactions, network permeability, and pH-dependent transport mechanisms in ionically crosslinked alginate hydrogels.

Despite the growing number of studies on alginate-based drug delivery systems, comparative investigations evaluating structurally different antibiotics under identical formulation conditions remain limited. In particular, the influence of drug physicochemical properties on encapsulation efficiency, hydrogel microstructure, and pH-responsive release behavior has not been systematically examined using a statistically optimized experimental design. We hypothesized that the distinct molecular characteristics of AMOX and DOX would lead to different encapsulation behaviors and release profiles within the same calcium–alginate matrix, thereby providing further insight into structure–property relationships governing hydrogel-based drug delivery systems.

Beyond formulation optimization, kinetic modeling provides further insight into the dominant release mechanisms. Models such as zero-order, first-order, Higuchi, and Korsmeyer–Peppas models are widely used to describe diffusion-controlled transport in polymeric colloidal matrices [8]. Temperature-dependent kinetic analysis using the Arrhenius equation additionally offers a means to evaluate thermal sensitivity [13].

Therefore, this study aimed to optimize the encapsulation of AMOX and DOX in calcium–alginate hydrogel beads using a Box–Behnken experimental design and to elucidate their pH-responsive release behavior through kinetic modeling. Furthermore, comprehensive physicochemical characterization, including FTIR spectroscopy, optical microscopy, SEM, DLS, and zeta potential analyses, was performed to establish structure–property relationships between formulation parameters, hydrogel microstructure, and drug release behavior.

## 2. Results and Discussion

### 2.1. Encapsulation of AMOX/DOX-Loaded Alginate Beads

Table S1 shows that the EE of the AMOX-alginate beads changed between 10% and 86%. This wide distribution indicates that the process conditions affect the encapsulation yield significantly. The highest EE (86%) was obtained by Run 4 (3.5% alginate, 3% CaCl_2_, and 45 min of hardening time). The lowest EE (10–13%) was observed by the low-alginate or low-CaCl_2_ conditions (Runs 1 and 17). Encapsulation efficiency tends to increase with alginate and CaCl_2_ concentrations. This can be due to the fact that the network structure becomes tighter through strong ionic crosslinking, creating a protective barrier. This structure protects the bioactive compounds against oxidative and structural degradation [14]. Efficiency might decrease due to diffusion limitation or insufficient gel formation under conditions of very high alginate (>5%) or low CaCl_2_ (<1%). On the other hand, five replicates at the center points (3.5% alginate, 2% CaCl_2_, and 30 min) show consistent results (EE ≈ 49–54%) across replicates (Runs 5, 6, 8, 13, and 16). Our best results are similar to those of chitosan–alginate microcapsules, including AMOX produced by ionic gelation, which achieved 84% EE [15]. Khoshnood et al. reported 76% EE for the encapsulation of AMOX in docosahexaenoic acid-loaded chitosan/alginate nanoparticles [16]. Girigoswami (2023) observed 64% EE of AMOX trapped in chitosan–alginate nanohydrogels [17].

EE for DOX-loaded alginate beads varied from 10% to 63%, as seen in Table S2. The highest EE (63%) was achieved with a moderate alginate concentration (3.5%, *w*/*v*), a high CaCl_2_ concentration (3%, *w*/*v*), and a short gelation time (15 min). In contrast, low CaCl_2_ levels (1%, *w*/*v*) and prolonged gelation (45 min) generally resulted in reduced EE. DOX-loaded microcapsules based on type II collagen, sodium alginate, and sodium carboxymethyl cellulose yielded 67.57% and 80.14% EE [18]. The lower EE in the present study might be attributable to the simpler matrix system (sodium alginate) and the absence of additional polymers (type II collagen, sodium alginate, and sodium carboxymethyl cellulose). Similarly, Singh et al. reported 85% EE for DOX-loaded particles containing graphene oxide, chitosan, and alginate [19]. DOX-loaded PLA microspheres produced by the emulsion solvent evaporation technique gave 38% EE [20]. Recently, pH-sensitive microparticles of DOX yielded 69.43% ± 5.32 EE [21]. DOX-loaded electrospray-generated poly(lactic-co-glycolic acid) (PLGA) microspheres showed 40.8%, 46.6%, 58.6%, and 66.7% EE depending on the polymer end groups [22].

To sum up, both AMOX and DOX encapsulation efficiencies were primarily governed by alginate and calcium chloride concentrations, whereas the influence of gelation time differed between the two antibiotics. These results demonstrate that the physicochemical characteristics of the drug affect the optimal formulation conditions.

### 2.2. Statistical Analysis

ANOVA findings (Table S3) give the statistical validity of the Box–Behnken Design model created for AMOX encapsulation. The statistical results are quite favorable. F-value (12.90) and *p*-value (<0.0001) of the model show that the model is statistically significant. Considering R^2^ (0.9938), the model derived by the software (Equation (8)) explains 99.38% of the data. Since the difference between Adjusted R^2^ and Predicted R^2^ is less than 0.2, they agree and support the model’s high predictive power. Accordingly, coefficient of variation (C.V.) also shows that the experimental work is reliable, since less than 10% of the C.V. is considered satisfactory. A non-significant lack of fit also reflects the model’s ability to fit [23]. Furthermore, the five replicated center points (Runs 5, 6, 8, 13, and 16) exhibited only minor variation in encapsulation efficiency (38–42%), indicating excellent experimental repeatability. In addition, the difference between the Adjusted R^2^ (0.9678) and Predicted R^2^ (0.8204) was below the commonly accepted threshold of 0.20, supporting the predictive capability of the quadratic model without evidence of substantial overfitting. The adequacy of the model was further confirmed by the close agreement between the predicted encapsulation efficiency (61.77%) and the experimentally validated value (63.87%).

(1) $$EE=51.80+1.50A+20.50B-2.25C-0.25AB+2.75AC+5.25BC-26.78A^{2}+5.22B^{2}+3.23C^{2}$$

**Figure 1:**
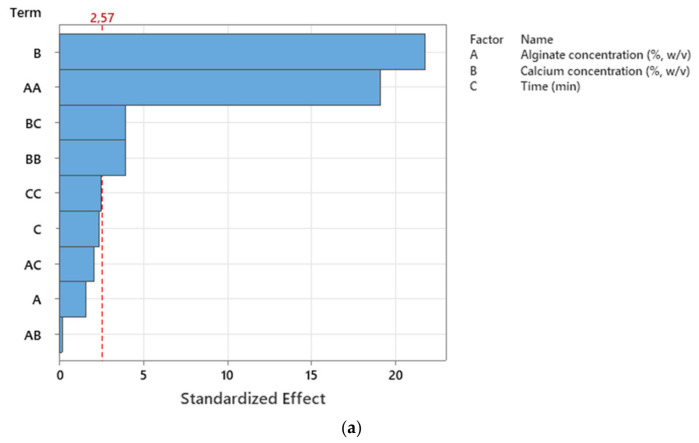

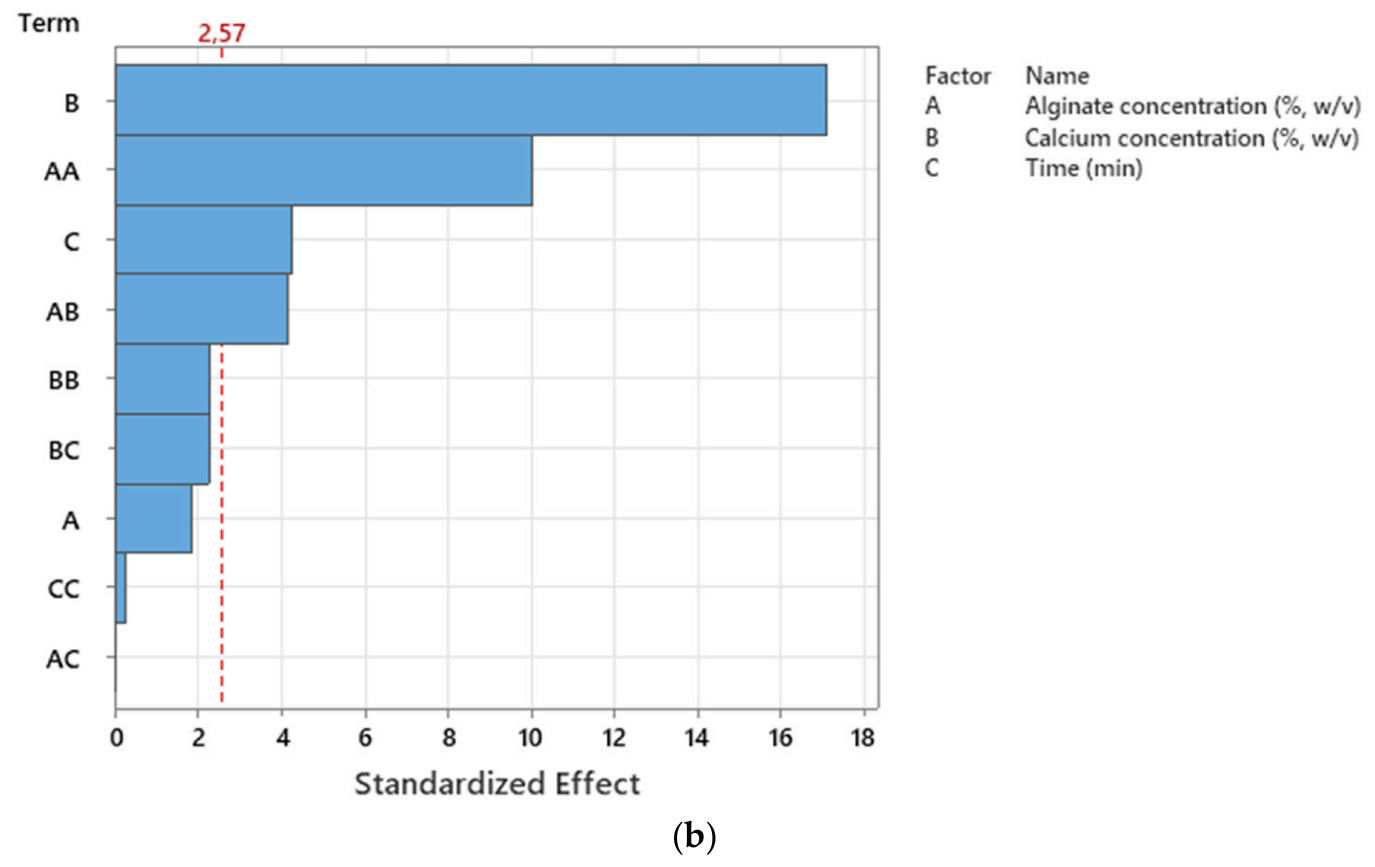
Pareto chart illustrating the significance of factors affecting the encapsulation efficiency of AMOX-loaded (**a**) and DOX-loaded (**b**) alginate beads.

Equation (8) shows that B (CaCl_2_) is the most dominant factor, while A (alginate) has a strong quadratic term (−26.78A^2^). On the other hand, a Pareto chart is the visual summary of effect sizes as given in Figure 1a. The red dashed line (2.57) in Figure 1a demonstrates the significance boundary at the 95% confidence level (α = 0.05). As already mentioned in Equation (8), B (CaCl_2_ concentration) exceeds the line by a significant margin as the most significant factor. Alginate squared is the second effective term, where the alginate effect is nonlinear. This is followed by BC (CaCl_2_ × time). The remaining terms (C, AC, A, AB) are below the significance level. These findings match the *p*-values given in Table S3.

Table S4 is the ANOVA table demonstrating the statistical validity of the Box–Behnken Design model created for DOX encapsulation. The statistical findings are very positive. The model’s F-value (54.45) and *p*-value (<0.0001) demonstrate that it is statistically significant. The software’s model (Equation (9)) explains 98.59% of the data, which is based on R^2^ (0.9859). The model has a strong predictive power because the difference between Adjusted R^2^ and Predicted R^2^ is less than 0.2. C.V. also shows that the experimental work is reliable because less than 10% of the C.V. is acceptable. A non-significant lack of fit also represents the adequacy of the [23,].

(2) $$EE=40+1.75A+16B-4C-5.50AB+0.00AC+3BC-13A^{2}+4B^{2}+0.5C^{2}\;\;$$

Calcium chloride concentration exhibited the most powerful effect on EE (F = 322.16, *p* < 0.0001), followed by the second power of alginate concentration (F = 111.93, *p* < 0.0001) and gelation time (F = 20.13, *p* = 0.0028). The interaction term between alginate and calcium is also significant, as seen in Figure 1b. B^2^, BC, AC, C^2^, and the linear term of alginate concentration (A) do not exceed the critical 2.57 value (Figure 1b), meaning that these terms are not statistically significant (α = 0.05).

Overall, the response surface models showed good statistical performance with significant model terms, non-significant lack-of-fit, and satisfactory predictive capability, supporting their suitability for optimization within the investigated design space.

### 2.3. Response Surface and Contour Plots of Encapsulation Efficiency

**Figure 2:**
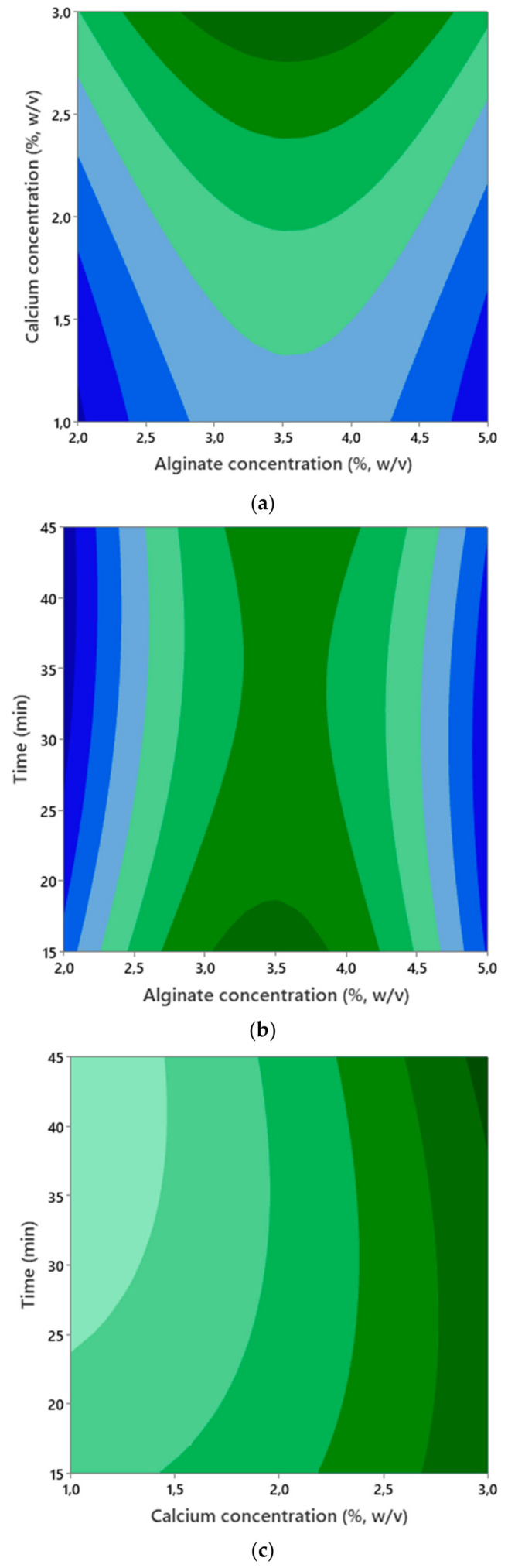
Contour plots showing the combined effects of alginate concentration and calcium chloride concentration (**a**), alginate concentration and time (**b**), and calcium chloride concentration and time (**c**) on encapsulation efficiency for AMOX-loaded alginate beads.

The combined effects of alginate, calcium chloride, and time on EE of AMOX-loaded alginate beads can be observed in the surface (Figure S1) and contour plots (Figure 2). The significance of the quadratic terms of alginate concentration and calcium chloride concentration (Table S3 and Figure 1a) can also be seen in Figure S1a, where there are curvatures on the surfaces. As seen in Figure S1a,b, increasing alginate concentration increased the encapsulation yield. This is because the number of crosslinks established with Ca^2+^ ions increases as the alginate concentration increases. This makes the gel stronger [2]. However, the diffusion of Ca^2+^ ions into the gel becomes difficult when the alginate is too dense [24]. Therefore, the EE started to decrease after around 3.5% of alginate concentration.

As the Ca^2+^ concentration increases, the rate of gel formation (crosslinking) increases (Figure S1c). The alginate chains bond more quickly, forming the gel [25]. However, the hardening time was not statistically significant, as already shown in the Pareto chart (Figure 1a). This non-significant effect on EE is shown in Figure S1b. Bennacef et al. reported that time (the period for the contact of alginate with calcium ions in the gelation bath) had no effect on the sphericity of the alginate beads [2]. On the other hand, BC interaction was significant, as seen in Figure 1. Therefore, increasing both time and calcium concentration enhances the efficiency of the encapsulation (Figure S1c).

It is seen that the yield reached a maximum at moderate alginate (3.5%) and high calcium chloride (3%) concentrations. The curvilinear structure (Figure 2) indicates that a quadratic optimum exists, especially for alginate. Extending the hardening time to 45 min increased EE. Dark green areas in Figure 2 indicate the optimum EE region above 80% (optimal area).

Figure 2 is consistent with the significant effect of the interaction between time and calcium concentration as given in (Table S3 and Figure 1a). The combination of high CaCl_2_ and long duration maximizes the yield.

**Figure 3:**
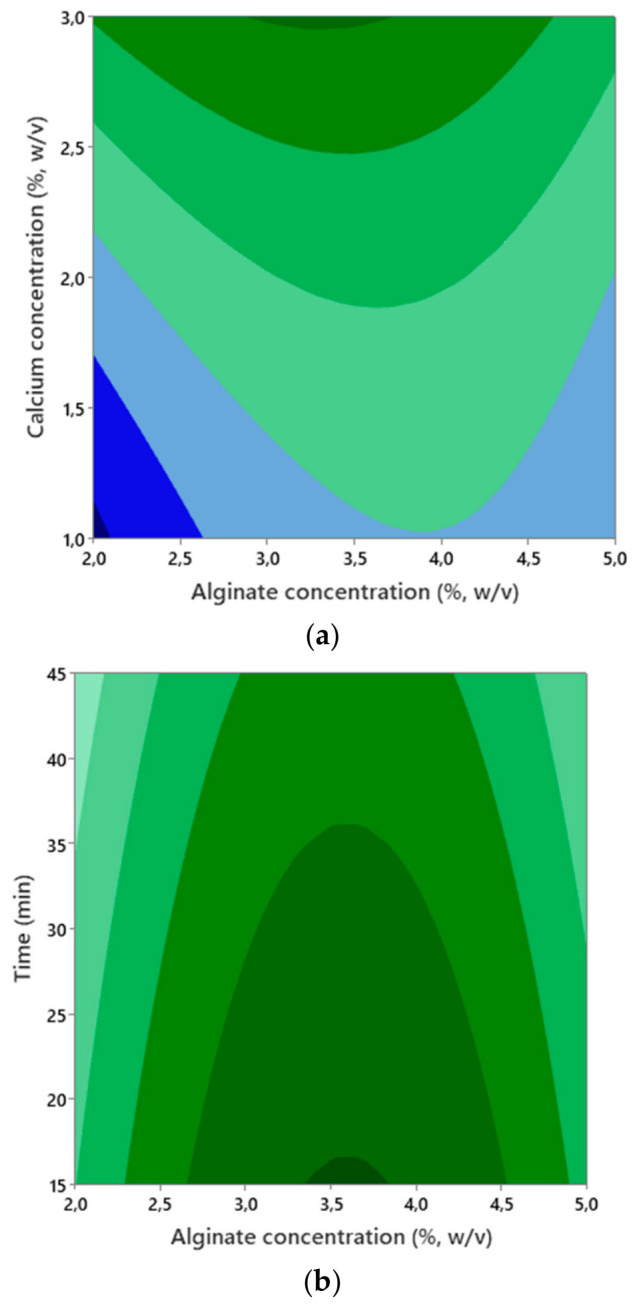

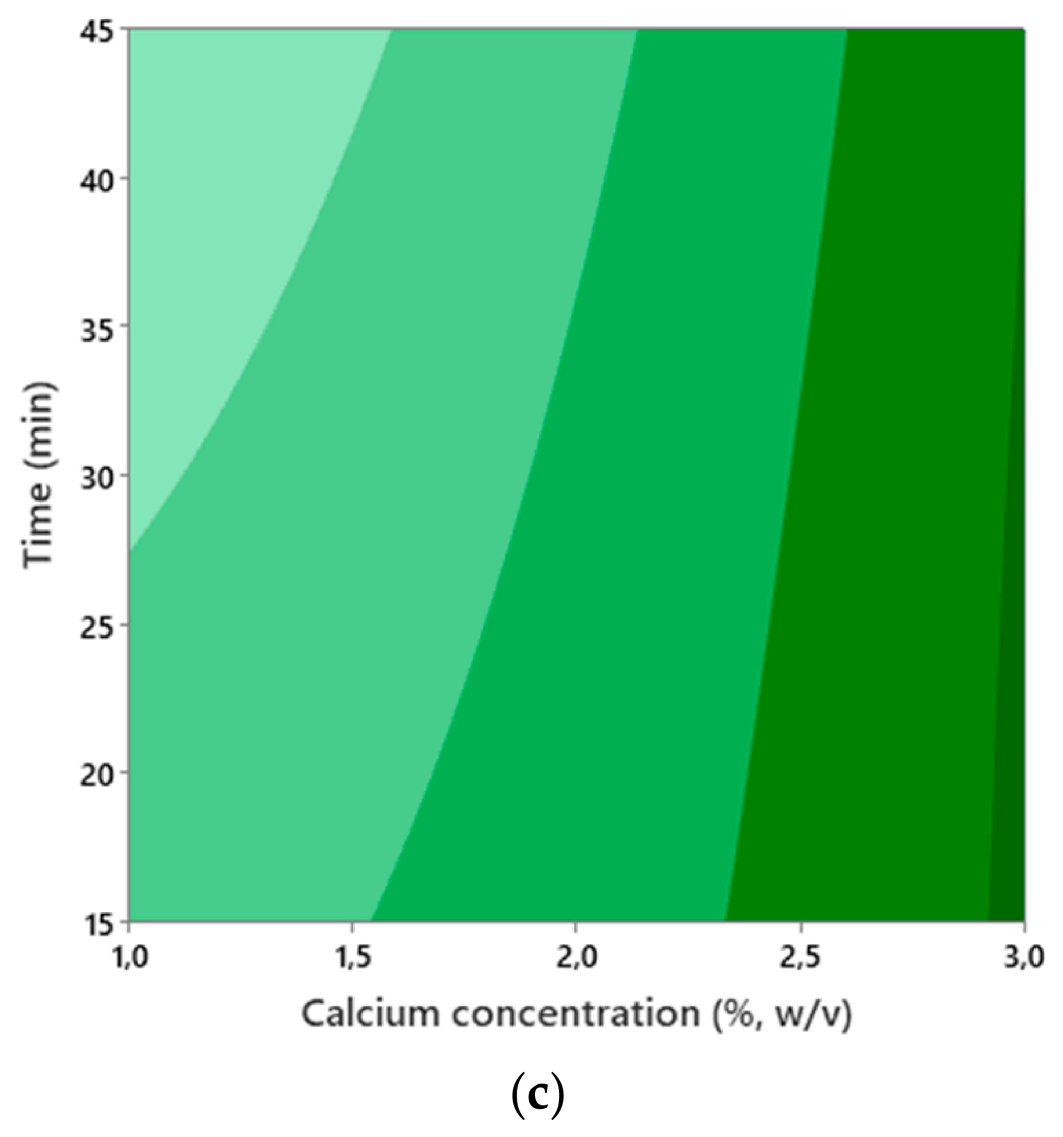
Contour plots showing the combined effects of alginate concentration and calcium chloride concentration (**a**), alginate concentration and time (**b**), and calcium chloride concentration and time (**c**) on encapsulation efficiency for DOX-loaded alginate beads.

The combined effects of alginate, calcium chloride, and time on EE of DOX-loaded alginate beads can be observed in the surface (Figure S2) and contour plots (Figure 3). Increasing alginate concentration causes a more viscous gel, leading to more binding sites. So, there is reduced diffusion loss as seen in Figure S2a,b. As the calcium concentration approaches 3%, the number of egg-box sites increases, resulting in a tighter crosslinked network. This reduces the escape of DOX from the capsule, while EE is increased (Figure S2a,c). The increase in EE due to an increase in alginate concentration is a common trend reported in the encapsulation of a wide variety of active substances (phenolic compounds, essential oils, plant extracts, and probiotic microorganisms. Alginate concentration, CaCl_2_ concentration and time were determined as significant process parameters in the Box–Behnken design [16,17]. Similarly, recent studies on the encapsulation of gallic acid, tangerine peel extract, fennel essential oil and probiotics in alginate-based systems have reported that alginate concentration, calcium concentration and/or gelation time control the encapsulation efficiency [7,24,26].

When the time is too long, excessive crosslinking occurs. Hence, slow diffusion loss of DOX due to the matrix hardening decreases the yield (Figure S2b). On the other hand, Figure S2c shows that EE increases linearly with the calcium concentration. Additionally, EE is observed to increase continuously over time as well. This can be explained by the fact that the crosslinking ion (Ca^2+^) makes the network structure denser over time, facilitating the trapping of DOX in the alginate bead.

Contour plots (Figure 3) demonstrate that alginate concentration, CaCl_2_ concentration, and gelation time are the primary factors of encapsulation efficiency. Specifically, EE was observed to reach maximum values at alginate levels of around 3.5% and CaCl_2_ levels of ~3%.

Figure 3a shows that EE reaches its maximum (>60%) when the alginate concentration is around 3.5%, and the CaCl_2_ concentration is around 3.0%. Figure 3b demonstrates that the optimum alginate level is between 3.5 and 4.0%, while the time in the range of 15–20 min maximizes EE (>45%). Figure 3c shows that EE increases regularly with the CaCl_2_ concentration. A slight EE increase is observed as the time increases from 15 min to 45 min.

These results confirm that a moderate alginate concentration combined with a high calcium chloride concentration provides the most favorable encapsulation conditions for both antibiotics.

### 2.4. Numerical Optimization of AMOX/DOX-Loaded Alginate Beads

**Figure 4:**
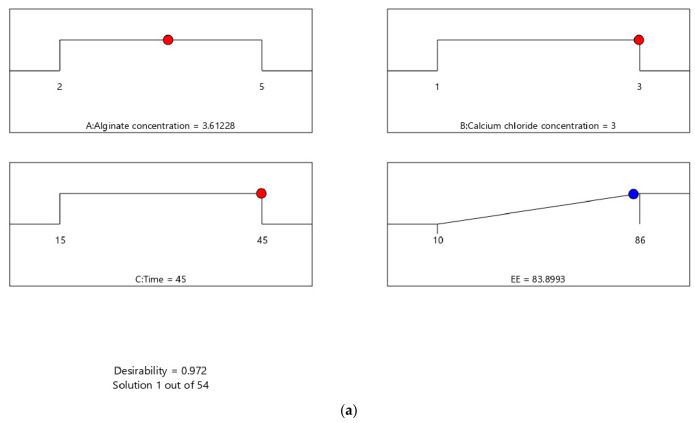

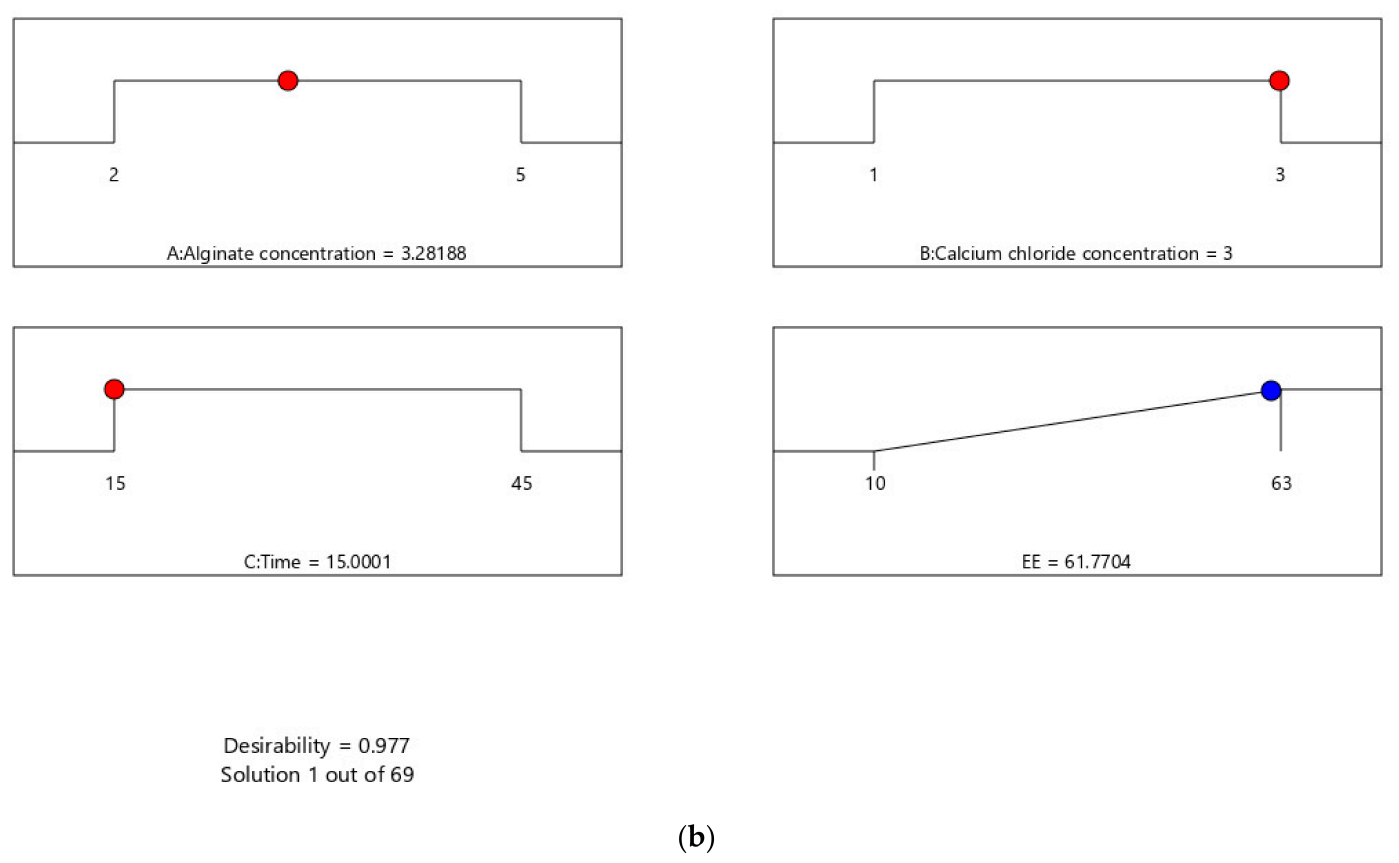
Ramps plot for optimization of encapsulation efficiency of AMOX-loaded (**a**) and DOX-loaded (**b**) alginate beads.

As a result of multiple optimizations performed using RSM, Design-Expert V13 software generated 56 possible solutions for the AMOX-loaded alginate beads. Among these solutions, the optimum condition with the highest desirability value was determined as given in the Ramps diagram (Figure 4a). To confirm these results, validation tests were performed under the proposed conditions. The experimental EE (~80%) was consistent with the predicted EE (83.90%), showing that the optimization model is accurate and reliable.

The independent factors (alginate concentration, calcium concentration, and gelation time) were kept within the experimental range to maximize the response function. In the case of DOX-loaded alginate beads, a total of 69 possible solutions were obtained in the desirability-based optimization performed by Design-Expert software, where the optimum conditions were 3.28% alginate, 3% calcium, and 15 min gelation time. Under these conditions, the predicted EE value was 61.77%. This finding can be seen in the Ramps plot (Figure 4b), where the desirability curves for all three factors point to the same optimum region; the system is optimized with high reliability. The actual EE value was determined to be 63.87% in the validation experiment using optimal conditions. This value is close to the model’s estimation (61.77%). This finding confirms the model’s accuracy.

The optimized formulations identified by the desirability function represent a balance between polymer availability and crosslinking density. Moderate alginate concentration provides sufficient polymer chains for drug entrapment without excessively increasing solution viscosity, whereas higher calcium chloride concentration promotes a denser hydrogel network through enhanced egg-box formation. Consequently, these conditions minimize drug diffusion into the gelation medium and maximize encapsulation efficiency.

On the other hand, the different optimum gelation times obtained for AMOX and DOX may also be interpreted from a polymer chemistry perspective. AMOX, owing to its hydrophilic nature, relies primarily on physical entrapment within the progressively densifying calcium–alginate network, making prolonged gelation beneficial for minimizing diffusion into the external gelation bath. In contrast, the amphiphilic DOX molecule contains multiple functional groups capable of hydrogen bonding and ionic interactions with alginate. These interactions likely facilitate earlier association with the polymer network during bead formation, so additional hardening contributes little to encapsulation while potentially allowing gradual redistribution or diffusion of weakly bound DOX into the gelation medium. Nevertheless, this interpretation remains hypothetical and would require further molecular-level investigation.

### 2.5. Characterization of AMOX/DOX-Loaded Alginate Beads

#### 2.5.1. FTIR Analysis

**Figure 5:**
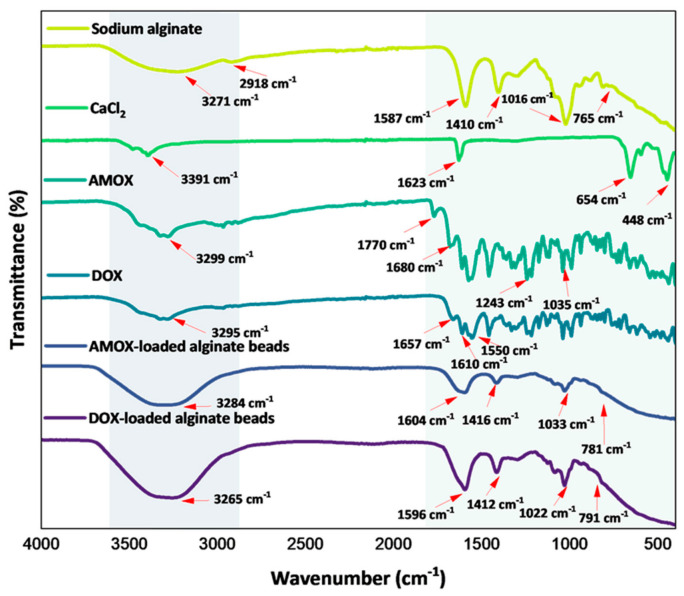
FTIR spectra of Sodium alginate, CaCl_2_, amoxicillin, doxycycline, amoxicillin-loaded alginate beads, and doxycycline-loaded alginate beads, respectively.

FTIR analysis was performed to better understand the interactions between the polymer matrix and the drugs, the associated changes in functional groups, and the molecular interactions occurring during the encapsulation process. In this context, the spectra of sodium alginate, CaCl_2_, pure AMOX, pure DOX, and drug-loaded alginate beads were compared (Figure 5). The combined evaluation of the spectra provides valuable information on both the structural changes in the alginate network after crosslinking and the spectral changes associated with drug incorporation into the matrix. The sodium alginate spectrum exhibits a broad O-H stretching vibration at 3271 cm^−1^, C-H stretching vibrations at 2918 cm^−1^, an asymmetric COO^−^ stretching band at 1587 cm^−1^, and a symmetric COO^−^ stretching band at 1410 cm^−1^ [21,22]. C-O-C vibrations around 1016 cm^−1^ and ring vibrations around 765 cm^−1^ confirm the polysaccharide backbone [23]. The wide band around 3391 cm^−1^ in the FTIR spectrum of CaCl_2_ shows the O-H stretching vibration of water molecules that are attached to the crystal structure in hydrated CaCl_2_. The band at 1623 cm^−1^ is linked to the H-O-H bending vibration, which shows that the salt is in its hydrated form [27]. The peaks at 654 and 448 cm^−1^ in the low wavenumber region are associated with Ca-O and Ca-Cl lattice stretching and deformation vibrations [7,28]. The FTIR spectrum of pure AMOX shows O-H/N-H stretching vibrations at 3299 cm^−1^ [29], the characteristic C=O band of the β-lactam ring at 1770 cm^−1^ [30], and the C=O vibration of the amide group at 1680 cm^−1^, consistent with the literature [24]. The bands at 1243 and 1035 cm^−1^ in the fingerprint region correspond to aromatic and aliphatic C-O and C-N vibrations of the drug [26]. The FTIR spectrum of DOX exhibits a prominent band at 3295 cm^−1^, signifying OH stretching vibration, in addition to amide bands I and II at 1610 cm^−1^ and 1550 cm^−1^, respectively. Also, the vibration at 1657 cm^−1^ is related to the stretching vibrations of the C=O and C=C groups in the DOX aromatic ring [31,32]. The FTIR spectrum of AMOX-loaded alginate beads contains distinct band shifts suggesting changes in the local chemical environment of the drug after encapsulation. Compared to pure AMOX, the shift and broadening of the O-H/N-H band at 3284 cm^−1^ are consistent with hydrogen-bonding interactions after loading. Indeed, the literature reports that similar shifts and broadening observed in the O-H/N-H bands when interactions occur between functional groups in polymer matrices are indicative of hydrogen bonding [33]. The appearance of the band at 1604 cm^−1^ suggests intermolecular interactions between the amide/carbonyl groups of AMOX and the COO^−^ groups of alginate. Additionally, the shift in the band at 1416 cm^−1^ indicates a shift in the symmetric COO^−^ stretching band, depending on the position of the drug in the Ca^2+^-crosslinked alginate network. The bands at 1033 cm^−1^ and 781 cm^−1^ in the fingerprint region support the physical retention of the drug within the matrix together with local conformational changes after loading [34]. The FTIR spectrum of DOX-loaded alginate beads, like that of AMOX-loaded alginate beads, contains distinct band shifts suggesting changes in the local chemical environment following encapsulation. Compared to pure DOX, the broadening of the O–H/N–H band around 3265 cm^−1^ is consistent with hydrogen-bonding interactions with the polymer chains [35]. Compared to the pure DOX spectrum, the shift of the band at 1596 cm^−1^ (C=O/amide I) suggests intermolecular interactions between the carbonyl/amide groups of DOX and the COO^−^ groups of alginate [36]. Similarly, the band around 1412 cm^−1^ indicates that the symmetric COO^−^ stretching band shifts depending on the position of DOX in the matrix. The preservation of the 1022 cm^−1^ and 791 cm^−1^ bands in the fingerprint region, on the other hand, supports the physical retention of DOX within the alginate network while preserving its molecular skeleton, together with local environmental changes after encapsulation [35].

#### 2.5.2. Morphological Analysis (SEM and Optical Microscopy)

Optical microscope images (Figure S3) show that alginate microcapsules obtained by ionic gelation, loaded with AMOX and DOX, generally have a homogeneous, uniform, and well-defined spherical morphology. AMOX capsules exhibit a lighter yellow and matte surface structure, while DOX capsules have a more transparent, densely pigmented, and smooth outer surface. A slight variation in the color intensity of the DOX-loaded beads (from light to dark yellow) was also observed. This is likely attributable to local differences in the distribution of doxycycline within the alginate matrix, together with optical effects arising from variations in bead thickness and light scattering, rather than differences in the preparation conditions. This difference might be related to the solubility of the drugs in the formulation, polymer–drug interactions, and the degree of crosslinking [37]. Size analyses reveal a narrow particle distribution for both drugs. The average diameter of AMOX-loaded alginate beads was found to be 1.97 ± 0.015 mm, while DOX-loaded alginate beads were 2.28 ± 0.018 mm. Spherical factor and roundness values indicate that both microcapsule groups exhibit a high degree of sphericity. SF value is 0.048 for the AMOX sample, whereas it is 0.037 for the DOX sample. These values close to zero, showing ideal sphericity [38]. Similarly, the roundness values (AMOX: 1.505 and DOX: 1.175) confirm good geometric integrity and minimal deformation in both systems.

**Figure 6:**
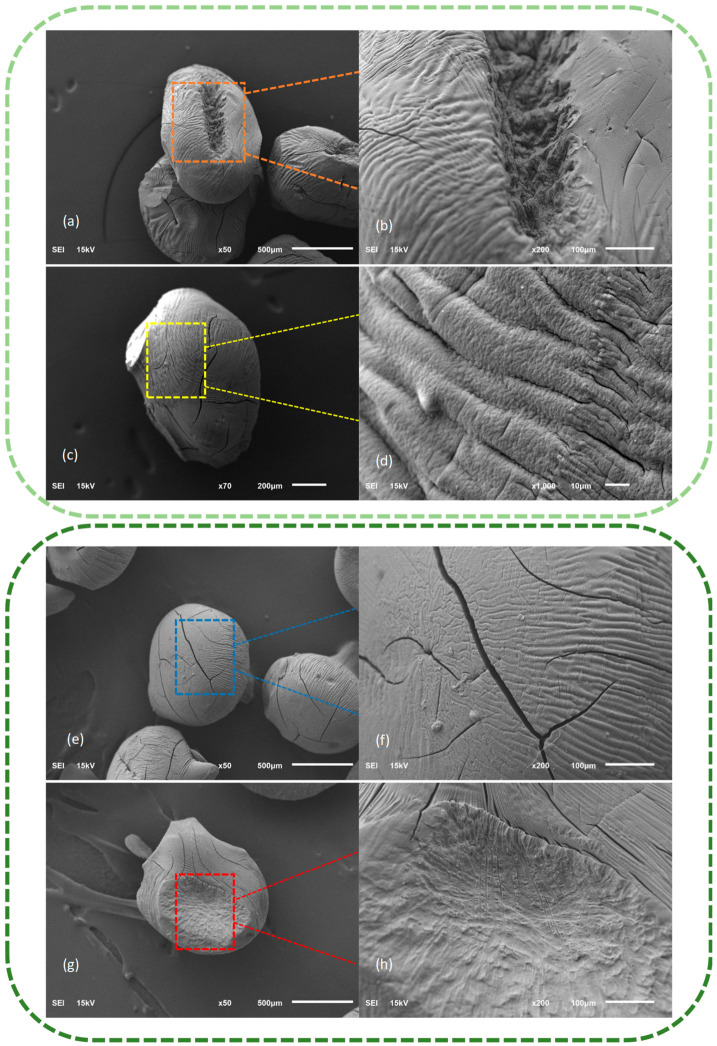
SEM images of AMOX-loaded (**a**–**d**) and DOX-loaded (**e**–**h**) alginate beads.

Figure 6 shows the surface topography of alginate beads loaded with AMOX (a–d) and DOX (e–h). Both formulations exhibit a general spherical/oval macro structure, indicating successful encapsulation, and display a wrinkled surface topography resulting from the drying of the beads. This wrinkled structure is a typical result of rapid water loss and shrinkage experienced by hydrogel matrices during drying, which has been widely reported in alginate-based carrier systems [39,40]. AMOX-loaded beads exhibit a structure with dense, parallel, and regular curls on the surface (Figure 6d, ×1000). This wavy appearance at high magnification indicates that hydrophilic drugs such as AMOX are homogeneously distributed within the alginate matrix. The morphology of DOX-loaded beads shows some differences from AMOX beads. While the curled main structure is preserved, the long, linear surface cracks seen particularly in Figure 6f and the localized rough/irregular areas in Figure 6h are noteworthy. Such structural defects are attributed to the amphiphilic nature of the DOX molecule. It is thought that amphiphilic drugs can cause heterogeneous aggregations and localized microstress regions in the polymer network by forming both hydrogen bonds and hydrophobic interactions with alginate chains [41]. These findings clearly demonstrate the different effects that two distinct drug molecules have on the microstructure of the alginate matrix.

#### 2.5.3. DLS and Zeta Potential Measurements

**Figure 7:**
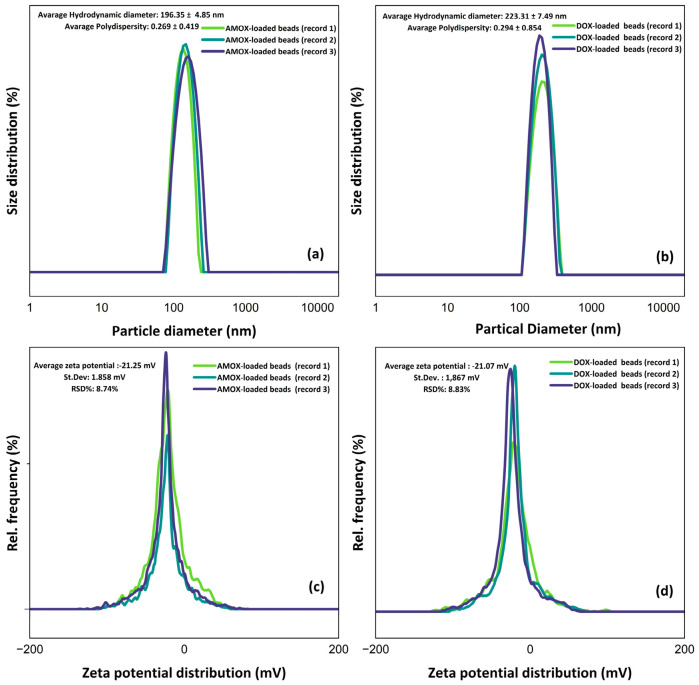
Hydrodynamic particle size distributions (**a**,**b**) and zeta potential (**c**,**d**) profiles of the released fraction collected from AMOX-loaded and DOX-loaded beads following incubation in phosphate-buffered saline (pH 6.8). Measurements represent three independent replicates (records 1–3). Data correspond to particles and molecular complexes present in the supernatant after drug release.

After incubation of AMOX-loaded and DOX-loaded beads in phosphate-buffered saline (pH 6.8), measurements of the hydrodynamic size and zeta potential of the released fraction (supernatant) were performed. Therefore, the obtained DLS and zeta potential data correspond to the nanoscale particles and molecular complexes present in the supernatant after release. Upon examination of Figure 7a,b, it is observed that the fraction released from AMOX-loaded beads has an average hydrodynamic diameter of 196.35 ± 4.85 nm and a low polydispersity value (0.269 ± 0.419). Similarly, the supernatant fraction obtained from DOX-loaded beads showed a slightly higher average size (223.31 ± 7.49 nm) and a similar polydispersity value (0.249 ± 0.854). PDI values below 0.3 indicate that the released fraction has a relatively monodisperse distribution and that the particles are homogeneously suspended [42]. The zeta potential results (Figure 7c,d) revealed that the AMOX-released fraction carried an average surface charge of −21.25 mV, while the DOX-released fraction carried −21.07 mV. The measured zeta potentials fall within the range typically interpreted as moderate colloidal stability, suggesting that the released species remain adequately electrostatically stabilized in both systems [43]. In addition, the low variation between replicates (RSD% < 9) reflects the consistency and reliability of the measurements. Taken together, these results indicate that the materials released from both bead formulations maintain stable hydrodynamic sizes and surface characteristics in the supernatant.

The nanoscale species detected in the release medium most likely originate from the swelling and partial relaxation of the calcium–alginate network under the release conditions. This process may facilitate the release of small alginate-rich fragments together with drug-associated polymeric assemblies into the supernatant [44,45]. Such assemblies may include drug–polymer molecular complexes formed through non-covalent interactions between dissolved alginate chains and released drug molecules, as reported for similar hydrogel-based delivery systems [46,47,48]. Therefore, the particle populations detected by DLS are considered to represent released nanoscale species rather than intact hydrogel beads. Nevertheless, since DLS provides information only on hydrodynamic size rather than structural identity, the exact nature of these species cannot be conclusively determined based on the present data alone [49].

Overall, the characterization results consistently demonstrated successful encapsulation while revealing drug-dependent differences in microstructure and molecular environment within the alginate matrix.

### 2.6. Kinetic Modeling of Drug Release

Table 1 shows the kinetic analysis of drug release from alginate-based beads into SGF and SIF. SGF represents the simulated gastric fluid at around pH 1.2. First of all, the first-order kinetic modeling of AMOX release behavior in SGF produced a low R^2^ (≈0.5). This finding points out that the release rate was not governed by the remaining drug concentration. The release kinetics in the SGF fit the zero-order kinetic model moderately (R^2^ = 0.82), while the Higuchi model provided the best fit (R^2^ = 0.95). This result is consistent with the diffusion-controlled release from a hydrated polymer matrix (alginate) even in an acidic medium, where it is expected for matrix-controlled systems as described by Higuchi [50]. Onuigbo et al. also reported that the release kinetics of the AMOX encapsulated in mucoadhesive alginate-coated chitosan microparticles were better described by the Higuchi model rather than by zero-order, first-order, and Korsmeyer–Peppas kinetic models in both SGF and SIF [51].

SIF presents the simulated intestinal fluid at near-neutral pH. The AMOX release kinetics in SIF followed all models adequately. On the other hand, the kinetic data fit the Higuchi model (R^2^ = 0.94) and the first-order kinetic model (R^2^ = 0.87) much better, which implies the diffusion as the dominant mechanism. The higher k_H_ value in SIF (0.0095 mg min^−1/2^ versus 0.0352 mg min^−1/2^) means faster release from the stomach. These findings are consistent with earlier research in which drug diffusion (especially hydrophilic actives) is predominant [52,53]. Philip et al. also reported that diffusion was predominant in drug release with the increased k_H_ by pH [54]. Patil et al. also reported Higuchi fit in pure alginate systems, while chitosan–alginate followed zero-order kinetic release [55]. They also observed that the release rate increased with increasing pH, pointing to a more rapid and consistent release in SIF (zero-order kinetic model). Similarly, the higher k_0_ (almost four times) in SIF (0.0020 mg·min^−1^ versus 0.0005 mg·min^−1^) supports the pH-responsive behavior of the alginate matrix proposed in the present study. Furthermore, the Korsmeyer–Peppas model was also applied to the data points exhibiting less than 60% release [56]. Because 0.43 = n corresponds to a Fickian diffusion mechanism, 0.43 > n corresponds to a quasi-Fickian diffusion mechanism, and 0.43 < n < 0.89 corresponds to non-Fickian transport [57,58]. A Korsmeyer–Peppas analysis supports the kinetic findings. The n value (0.498) of the SGF condition (k = 0.0117 min^−n^ and R^2^ = 0.93) denotes non-Fickian transport (AMOX diffusion and network swelling) [59,60]. The n value of the SIF condition is below 0.43 (k = 0.159 min^−n^ and R^2^ = 0.99), corresponding to a quasi-Fickian diffusion mechanism. Diffusion is dominant, which means that the diffusion rate parameter increases as the pH increases.

Considering the DOX release behavior, R^2^ is very poor (Table 1), while mechanistic interpretation should be cautious (R^2^ ≤ 0.58). The relatively poor correlation with the conventional kinetic models suggests that DOX release cannot be adequately described by a single dominant mechanism. Instead, the release is likely governed by the simultaneous contribution of hydrogel swelling, polymer relaxation, ion exchange, and drug–matrix interactions, resulting in non-ideal release kinetics that are not fully captured by the classical kinetic models [47,61,62]. In the case of the SIF environment, the release kinetics are represented adequately by the Higuchi and first-order kinetic models. The Peppas analysis yielded an n lower than 0.43 with strong linearity only in SIF (R^2^ = 0.98), pointing to a quasi-Fickian diffusion mechanism as the controlling mode for the DOX release in the intestine. This diffusion-controlled behavior is also supported by the R^2^ values of the Higuchi model, which are higher than the others [63,64]. Furthermore, k_H_ is approximately 3.4 times higher in the intestine. This is because the pores are enlarged as a result of alginate swelling and ion exchange with increasing pH [65].

On the other hand, the release behavior of the AMOX and DOX is quite different even though they are trapped in the same alginate matrix. This can be explained by the physicochemical properties of these two drugs [66]. The superior release performance of AMOX over DOX can be attributed to its hydrophilic structure and weaker ionic complexation with the alginate matrix owing to its different pKa value.

To conclude, the release studies demonstrate that both antibiotics exhibited pH-responsive and predominantly diffusion-controlled release, although the release behavior depended on their physicochemical properties.

## 3. Conclusions

Alginate beads have been developed as a promising matrix for the encapsulation and controlled release of two structurally different drugs (amoxicillin and doxycycline). Box–Behnken response surface methodology showed that calcium chloride concentration and the quadratic effect of alginate concentration were the statistically significant factors. Under optimized conditions, high encapsulation efficiencies were achieved for both drugs (≈80–86% for AMOX and ≈62–64% for DOX). The different optimum gelation times obtained for AMOX and DOX are likely associated with their distinct physicochemical properties, although further investigation using intermediate gelation times may provide a more refined optimization of this parameter. Kinetic modeling in simulated gastric and intestinal fluids revealed that drug release from alginate beads is diffusion-controlled and pH-responsive due to the fact that Higuchi and Korsmeyer–Peppas models provided the best fit (especially under intestinal conditions). The results demonstrate that the developed alginate beads exhibit pH-responsive release behavior characterized by limited drug release under simulated gastric conditions and enhanced release under simulated intestinal conditions. These findings suggest their potential as oral delivery systems providing stomach protection and preferential intestinal drug release under in vitro conditions. However, further in vivo investigations, such as gastrointestinal transit, mucoadhesion, intestinal retention, and antibacterial efficacy studies, are required to confirm these properties. FTIR, SEM and DLS analyses supported these findings, providing evidence consistent with drug–polymer interactions, characteristic microstructural features and the colloidal stability of the released fraction. These findings form the basis for further formulation refinement and in vivo evaluation.

## 4. Materials and Methods

### 4.1. Materials

Model drugs (amoxicillin trihydrate (≥98%) and doxycycline hyclate (≥98%)) were provided by Tokyo Chemical Industry (Tokyo, Japan). Phosphate-buffered saline, sodium alginate as the biopolymeric matrix, and calcium chloride as the crosslinker were purchased from Sigma-Aldrich (St. Louis, MO, USA). According to the manufacturer’s specifications, the sodium alginate exhibited a viscosity of 20–400 cP for a 1% aqueous solution. In addition, Zahoor et al. [67] reported the same commercial sodium alginate grade as a medium-viscosity alginate (approximately 3500 cP for a 2% *w*/*v* solution). Since sodium alginate is a naturally derived polydisperse polymer, an exact molecular weight is not specified by the manufacturer.

### 4.2. Preparation of Drug-Loaded Alginate Beads

Sodium alginate (2–5%, *w*/*v*) was dissolved in deionized water by magnetic stirring at 600 rpm for 2 h. AMOX or DOX was added to the alginate solution at a constant drug concentration of 0.5% (*w*/*v*). Accordingly, the drug-to-alginate mass ratios were 1:4, 1:7, and 1:10 for alginate concentrations of 2.0%, 3.5%, and 5.0% (*w*/*v*), respectively. These ratios therefore varied only because of the alginate concentration selected in the experimental design and were not treated as an independent experimental factor. All formulations were prepared immediately prior to gelation. AMOX/DOX-loaded alginate solutions were dropped into the gelling medium (1–3%, *w*/*v*) with a syringe driver (New Era Pump Systems, Inc., Farmingdale, NY, USA) under gentle stirring (275 rpm). Rapid ionic crosslinking between Ca^2+^ and the guluronic acid blocks of alginate resulted in bead formation following the egg-box mechanism. The beads were allowed to harden for 15–45 min depending on the experimental design. After the gelation was completed, the alginate beads were filtered and washed with deionized water. Then, the beads were dried at ambient conditions. After washing, the beads were dried under identical ambient laboratory conditions until no visible surface moisture remained. The same drying procedure was applied to all formulations prior to further characterization and release experiments.

### 4.3. Determination of Encapsulation Efficiency

The encapsulated AMOX/DOX was extracted by dissolving the beads in phosphate-buffered saline using a homogenizator (IKA T25, ULTRA-TURRAX, Staufen, Germany). A spectrophotometer (PG Instruments, T60/Leicestershire, Leicestershire, England, UK) was used to quantify the drug concentration at 272 nm (AMOX) and 275 nm (DOX), respectively. The encapsulation efficiency (EE) was assessed by quantifying the drug concentration in the dispersion medium. EE was calculated as follows [68]:

(3) $$EE\;(\%)=\frac{W_{1}-W_{2}}{W_{1}}\times 100$$

*W*_1_ is the total amount of drug that was used in the mixture, while *W*_2_ is the amount of free drug that was measured in the supernatant. The amount of non-encapsulated drug (*W*_2_) was determined from the gelation medium remaining after bead formation. Following gelation, the beads were rapidly collected by filtration and briefly rinsed with a small volume of deionized water to remove residual calcium chloride. Since the washing step was performed rapidly using a minimal amount of water, drug loss during washing was assumed to be negligible and was therefore not included separately in the encapsulation efficiency calculation.

### 4.4. Characterization of Alginate Beads

The alginate beads produced under the optimal ionic gelation conditions were subjected to a CMOS-based digital imaging/camera system (Cameram 5, SOIF Optical Instruments, Shanghai, China) attached to an optical microscope. The camera has a 5-megapixel sensor (2592 × 1944 px) and is equipped with built-in analysis software. It enables dimensional measurements (d_min_, d_max_, area, and perimeter) and morphological evaluation (roundness and sphericity factor) [67]:

(4) $$Sphericty\;factor\;\left(SF\right)=\;\frac{d_{max}-d_{min}}{d_{max}+d_{min}}$$

(5) $$Roundness\;(Rn)=\frac{P^{2}}{4\pi A}$$

The further characterization of alginate beads was performed using Fourier Transform Infrared Spectroscopy (FTIR), Scanning Electron Microscopy (SEM), and Dynamic Light Scattering (DLS) analyses, including particle size, polydispersity index (PDI), and zeta potential measurements.

FTIR measurements were performed using a Bruker FTIR spectrometer (Bruker, Tensor 27, Ettlingen, Germany) in the range of 4000–400 cm^−1^. The surface morphology and microstructural properties of the beads were examined using a JEOL scanning electron microscope (JEOL, JSM-6610, Tokyo, Japan) after applying a thin gold coating to ensure conductivity prior to analysis. Measurements of particle size distribution, PDI, and zeta potential were executed utilizing an Anton Paar DLS apparatus (Anton Paar, Litesizer 500, Graz, Austria). DLS and zeta potential measurements were carried out at 25 °C. Samples were suitably diluted to guarantee measurement precision.

### 4.5. Experimental Design and Statistical Modeling

Design-Expert software (12.0.1.0) was used to design the experimental study based on a three-factor and three-level Box–Behnken design. The selection of sodium alginate concentration, calcium chloride concentration, and gelation time as the independent variables was based on both preliminary experiments and previous studies demonstrating that these parameters are the primary factors governing calcium–alginate bead formation, crosslinking density, encapsulation efficiency, and drug release behavior [59,61,63,64]. Other processing parameters (dispensing rate, needle diameter, stirring speed, preparation conditions, and drug concentration) were kept constant throughout all experiments. The drug concentration was fixed at 0.5% (*w*/*v*); therefore, the corresponding drug-to-alginate ratio changed only with alginate concentration and was not included as an independent variable in the Box–Behnken design.

In total, seventeen runs with five center points were produced by the software. Alginate concentration (A, 2–5%, *w*/*v*), CaCl_2_ concentration (B, 1–3%, *w*/*v*) and gelation time (C, 15–45 min) were the independent variables, while EE was the response. Analysis of variance (ANOVA) was also performed by Design-Expert software to evaluate the model’s significance, lack of fit, and coefficient of determination (R^2^; Adjusted R^2^ and Predicted R^2^). Furthermore, Minitab statistical software 22 (Minitab Inc., State College, PA, USA) was used to generate Pareto charts, response surfaces, and contour plots.

### 4.6. In Vitro Release Studies

The AMOX/DOX release was evaluated in simulated gastric fluid (SGF) at pH 1.2 and simulated intestinal fluid (SIF) at pH 6.8, respectively. Specified quantities of beads (0.2 g) were introduced into 50 mL of dissolution medium and incubated in conical flasks maintained at 37 °C under gentle agitation (100 rpm). At designated time intervals, aliquots were extracted, filtered, and quantified using spectrophotometry. An equivalent volume of fresh medium was replaced.

#### Kinetic Modeling

Zero-order (Equation (6)), first-order (Equation (7)), Higuchi (Equation (8)), and Korsmeyer–Peppas (Equation (9)) models were used to evaluate the release kinetic using linear regression equations below [69,70]:

(6) $$Q_{t}=Q_{0}+k_{0}t$$

$Q_{0}$ = Initial concentration of the drug in the solution (mg $g^{-1}$)

$Q_{t}$ = Concentration of the drug released at time t (mg $g^{-1}$)

$k_{0}$ = Zero-order release constant (mg $\min^{-1}$)

t = Time (min)

(7) $$\log C_{t}=\log C_{0}-k_{1}t$$

$C_{0}$ = Initial concentration of the drug in the microcapsules (mg $g^{-1}$)

$C_{t}$ = Concentration of the drug remaining in the microcapsules at time t (mg $g^{-1}$)

$k_{1}$ = First-order release constant ($\min^{-1}$)

(8) Qt=kHt12

$k_{H}$: Higuchi release constant (mg min−12)

(9) $$\frac{M_{t}}{M_{\infty }}=K\;t^{n}$$

$M_{t}$: Amount of drug released at time t (mg $g^{-1}$)

$M_{\infty }$: Total amount of drug released at infinite time (maximum release) (mg $g^{-1}$)

K: Release rate constant ($\min^{-n}$)

n: Release exponent indicating the drug release mechanism

## Figures and Tables

**Table 1:** Kinetic model parameters for the release of drug-actives from alginate beads.

Active Material	GI System	Zero-Order Kinetic Model		First-Order Kinetic Model		Higuchi Model		Korsmeyer–Peppas Model		
		k_0_ (mg min^−1^)	R^2^	k_1_ (min^−1^)	R^2^	k_H_ (mg min^−1/2^)	R^2^	n	K (min^−^^n^)	R^2^
AMOX	SGF	0.0005	0.8218	−0.0016	0.4803	0.0095	0.9548	0.4980	0.0117	0.9304
	SIF	0.0020	0.9393	0.0047	0.8698	0.0352	0.9446	0.2373	0.1590	0.9868
DOX	SGF	0.0004	0.3548	0.0006	0.3892	0.0086	0.4840	0.1203	0.2050	0.5831
	SIF	0.0014	0.5715	0.0038	0.7100	0.0291	0.7591	0.1388	1.2460	0.9789

## Data Availability

The datasets generated and/or analyzed during the current study are available from the corresponding author upon reasonable request.

## Supplementary Materials

The following supporting information can be downloaded at https://www.mdpi.com/article/10.3390/gels12070636/s1. Figure S1: Response surface plots showing the combined effects of alginate concentration and calcium chloride concentration (a), alginate concentration and time (b), and calcium chloride concentration and time (c) on encapsulation efficiency; Figure S2: Response surface plots showing the combined effects of alginate concentration and calcium chloride concentration (a), alginate concentration and time (b), and calcium chloride concentration and time (c) on encapsulation efficiency; Figure S3: Microphotographs of AMOX-loaded (a) and DOX-loaded (b) alginate beads. Scale bar = 1 mm; Table S1: Experimental results of the AMOX encapsulation in alginate beads based on the Box–Behnken design matrix; Table S2: Experimental results of the DOX encapsulation in alginate beads based on the Box–Behnken design matrix; Table S3: ANOVA results of the AMOX encapsulation in alginate beads; Table S4: ANOVA results of the DOX encapsulation in alginate beads.
