# Supplementary material for: Structure-Property Relationships Governing Encapsulation and Release of Antibiotics from Calcium–Alginate Hydrogels
Source: Gels. 2026 Jul 16;12(7):636. doi: 10.3390/gels12070636 (PMC13409666; doi:10.3390/gels12070636)
Supplement: Supplementary file 1 [file gels-12-00636-s001.zip › gels-4400814-supplementary.pdf]

Table S1. Experimental results of the AMOX encapsulation in alginate beads based on the design matrix of Box–Behnken.

Run	A: Alginate concentration (%, w/v)	B: Calcium chloride concentration (%, w/v)	C: Time (min)	EE (%)
1	2.0	1	30	10
2	5.0	2	15	30
3	2.0	3	30	48
4	3.5	3	45	86
5	3.5	2	30	53
6	3.5	2	30	51
7	3.5	1	45	31
8	3.5	2	30	49
9	5.0	2	45	30
10	5.0	3	30	50
11	2.0	2	15	32
12	3.5	3	15	79
13	3.5	2	30	52
14	3.5	1	15	45
15	2.0	2	45	21
16	3.5	2	30	54
17	5	1	30	13

Table S2. Experimental results of the DOX encapsulation in alginate beads based on the design matrix of Box–Behnken.

Run	A: Alginate concentration (%, w/v)	B: Calcium chloride concentration (%, w/v)	C: Time (min)	EE (%)
1	2.0	1	30	10
2	5.0	2	15	34
3	2.0	3	30	49
4	3.5	3	45	62
5	3.5	2	30	41
6	3.5	2	30	39
7	3.5	1	45	20
8	3.5	2	30	38
9	5.0	2	45	25
10	5.0	3	30	41
11	2.0	2	15	30
12	3.5	3	15	63
13	3.5	2	30	42
14	3.5	1	15	33
15	2.0	2	45	21
16	3.5	2	30	40
17	5	1	30	24

Table S3. ANOVA results of the AMOX encapsulation in alginate beads.

Source	Sum of Squares	df	Mean Square	F-value	p-value	
**Model**	6658.67	9	739.85	123.90	< 0.0001	significant
A-Alginate concentration	18.00	1	18.00	3.01	0.1261	
B-Calcium chloride concentration	3362.00	1	3362.00	563.01	< 0.0001	
C-Time	40.50	1	40.50	6.78	0.0352	
AB	0.2500	1	0.2500	0.0419	0.8437	
AC	30.25	1	30.25	5.07	0.0591	
BC	110.25	1	110.25	18.46	0.0036	
A^2	3018.53	1	3018.53	505.50	< 0.0001	
B^2	114.95	1	114.95	19.25	0.0032	
C^2	43.79	1	43.79	7.33	0.0303	
**Residual**	41.80	7	5.97			
Lack of Fit	27.00	3	9.00	2.43	0.2052	not significant
Pure Error	14.80	4	3.70			
**Cor Total**	6700.47	16				
R^2=0.9938 Adjusted R^2=0.9857 Predicted R^2=0.9321 C.V.=5.66%						

Table S4. ANOVA results of the DOX encapsulation in alginate beads.

Source	Sum of Squares	df	Mean Square	F-value	p-value	
**Model**	3115.50	9	346.17	54.45	< 0.0001	significant
A-Alginate concentration	24.50	1	24.50	3.85	0.0904	
B-Calcium chloride concentration	2048.00	1	2048.00	322.16	< 0.0001	
C-Time	128.00	1	128.00	20.13	0.0028	
AB	121.00	1	121.00	19.03	0.0033	
AC	0.0000	1	0.0000	0.0000	1.0000	
BC	36.00	1	36.00	5.66	0.0489	
A^2	711.58	1	711.58	111.93	< 0.0001	
B^2	67.37	1	67.37	10.60	0.0140	
C^2	1.05	1	1.05	0.1656	0.6962	
**Residual**	44.50	7	6.36			
Lack of Fit	34.50	3	11.50	4.60	0.0873	not significant
Pure Error	10.00	4	2.50			
**Cor Total**	3160.00	16				
R^2=0.9859 Adjusted R^2=0.9678 Predicted R^2=0.8204 C.V.=7%						

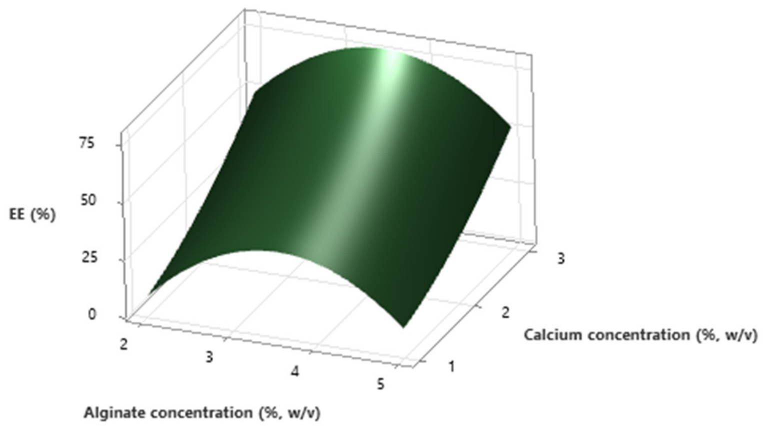
EE (%)
75
50
25
0
2
3
4
5
Alginate concentration (%, w/v)
1
2
3
Calcium concentration (%, w/v)

a

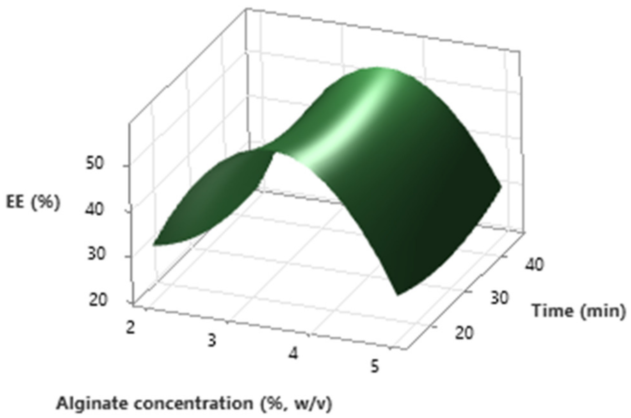
EE (%)
50
40
30
20
2
3
4
5
Alginate concentration (%, w/v)
20
30
40
Time (min)

b

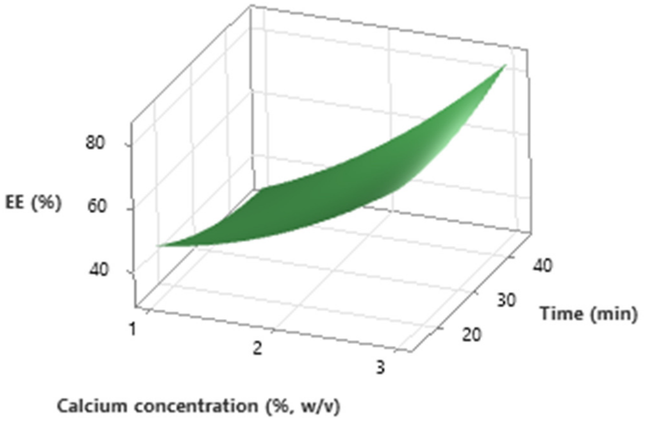

c

Figure S1. Response surface plots showing the combined effects of alginate concentration and calcium chloride concentration (**a**), alginate concentration and time (**b**), and calcium chloride concentration and time (**c**) on encapsulation efficiency.

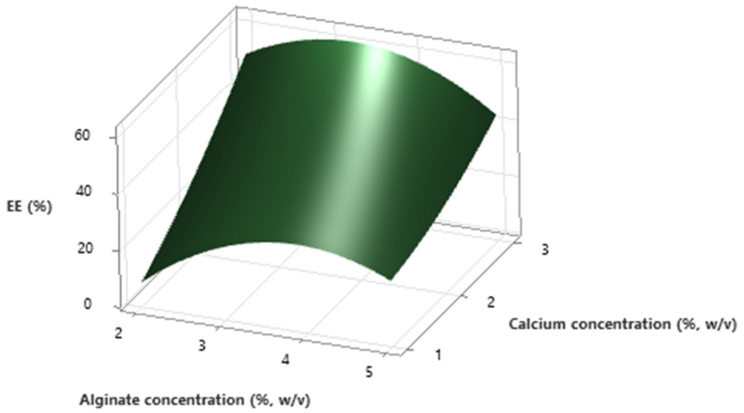

a

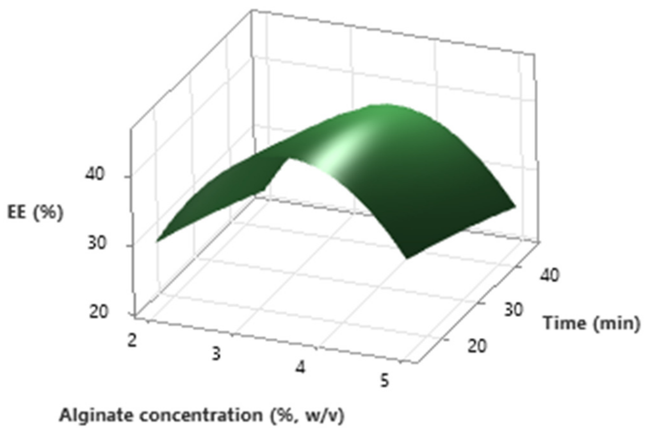

b

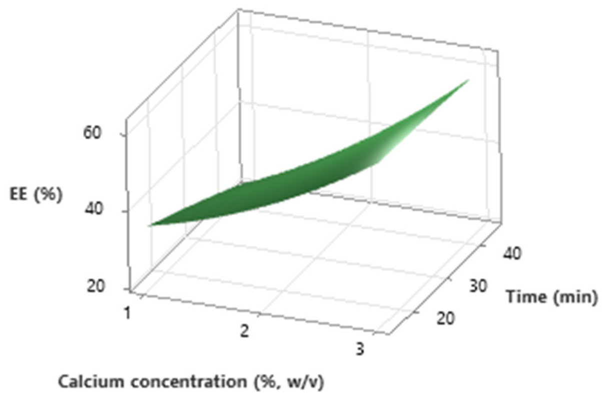

c

Figure S2. Response surface plots showing the combined effects of alginate concentration and calcium chloride concentration (**a**), alginate concentration and time (**b**), and calcium chloride concentration and time (**c**) on encapsulation efficiency.

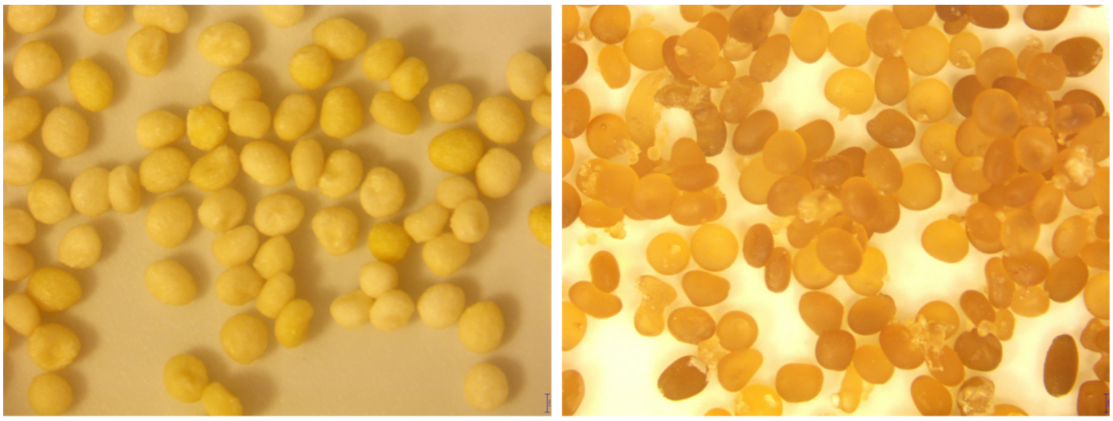

a) **b)**

Figure S3. Microphotographs of AMOX-loaded (**a**) and DOX-loaded (**b**) alginate beads. Scale bar = 1 mm.
